# The Great War: VI. The Progress of the Sick

**Published:** 1918-04-20

**Authors:** 


### THE GREAT WAR.

### VI.—THE PROGRESS OF THE SICK.

The tendency among the troops in the area of active operations is to delay reporting sick until they are so ill that they cannot any longer carry on. The moral of all ranks is so high that in some units it is a tradition that no man ever walks from the trenches to hospital; he sticks to his post as long as he can stand, and is then carried back. This has the disadvantage of causing delay in his recovery, a misfortune which is, however, more than counterbalanced by its effect in maintaining the bulldog tenacity for which our race is notorious. When, after their eight or more days in the trenches, the men come out into reserve, they seize the opportunity to consider their complaints, or are captured by the regimental medical officer at his health inspections.

The fate of the sick man after he has passed the portals of the regimental aid post varies considerably in the divisions. Both regimental and divisional commanders view with concern the loss of a single man from sickness; they have learned by experience that those who find their way to the base seldom return to their own battalions. Their places must be filled at once by strangers, and the recovered sick must be drafted from the depôt to the units most in need of them.

Consequently we find in some divisions a considerable harbouring of the milder forms of sickness, both in the battalions and in the field ambulances. Ordinarily the sick, with the exception of the most trivial cases, are evacuated daily from regimental aid posts to field ambulances.

### The Field Ambulance.

A field ambulance is a mobile unit having accommodation for 150 patients on palliasses. It consists of a "tent" division with a *personnel* of sixty-nine, including twenty-one trained in nursing duties, and a "bearer" division having a *personnel* of 114. In addition there are eight medical officers and a quartermaster, and drivers for nine motor ambulances and the horsed transport. Field ambulances are divisible into three independent sections, each with "tent" and "bearer" subdivisions.

This organisation suggests the purposes of field ambulances—the collection and removal of wounded from the battlefield, and a temporary resting-place on their journey to the clearing stations, but the stationary character of warfare on the Western Front has rendered possible their enormous development. From 150 palliasses in bell tents they have expanded to any number up to 1,000, in large buildings or hutted camps, on spring and wire beds, and stretchers on trestles.

Some of them are used as hospitals for the milder forms of sickness; others form special institutions for the treatment of scabies and other parasitic skin diseases, and so-called "rest stations," which receive convalescents from the field ambulances and feed them up and amuse them before their return to the trenches. At the field ambulances the sick are sorted; acute cases are passed on as soon as possible to the clearing stations, infectious, eye, and dental cases are sent to special hospitals, and those remaining are gathered into the local fold; these consist mainly of the milder cases of trench fever, myalgia, boils, and scabies.

### The "Scabies Station."

Scabies is treated at the Front, and as the patients are not struck off the strength of their units it is essential that they should be cured as quickly as possible. The commonest and most successful plan is the detachment of a field ambulance from a division, which, instead of forming a "dressing station," acts as a "scabies station," which serves the several divisions of a corps.

Approximately 300 beds are required, and a large treatment-room, extensive hot baths, heating arrangements, disinfecting chamber, a large stock of pyjamas, and a laundry in which they can be washed.

There are great advantages in a fixed institution of this description; the staff becomes interested the treatment thorough, and the cure rapid. Many methods of treatment are practised, and "one-day cures" of sufficiently early uncomplicated cases are claimed in considerable percentages for each. The greatest reliance is still placed on the long-established hot bath and sulphur ointment. There was recently a boom in the sulphur vapour or Russian cabinet system, which although it alleviated the irritation and apparently cured, gave false security, as relapses in a fortnight were frequent.

Liq. calcis sulphurata and balsam of Peru also have had extensive trials, but they are irritating and not generally used.

Although scabies cannot be entirely avoided, its early detection and thorough treatment, such as is possible only in a specialised institution, are capable of greatly reducing absence from the trenches. Early cases can be cured in three days, whereas neglected ones which reached a special hospital at the Base required an average of more than a month.

The sulphur treatment in a general sense is known to every medical practitioner, but in a particular sense to very few, and it is a subject of such vast import to an army that the details cannot be too often repeated.

On the first day the patient is thoroughly massaged with soft soap for ten minutes, he is next steeped in a hot bath up to the neck for

☛ Previous articles appeared on Feb. 9 and 23, March 9 and 23, and April 6, p. 7.

**THE GREAT WAR**—*(continued)*.

fifteen minutes, and then scrubbed with a nail-brush in order to open up burrows and vesicles. After drying, he is thoroughly rubbed all over with sulphur ointment.

This rubbing is repeated twice daily for three days. On the fourth day a second bath is given, and the course is completed by the issue of sterilised clothing and kit.

### The "Rest Stations."

Plenty to eat, sufficient to drink, a good bed, games to play, books to read, a band, a theatre, a cinema, playing at work, and free cigarettes—such is a description of a rest station just beyond ordinary shell range on the Western Front. Rest stations except in the devastated areas, where they are entirely of huts and tents, are generally based on large buildings such as châteaux, schools, monasteries, etc., supplemented by huts and tents; they are either divisional or corps institutions, in either case the accommodation provided is for approximately 300 patients per division.

They receive convalescents after their treatment for ten or twelve days in field ambulances, and retain them for a further fourteen days, or in exceptional cases for twenty-one days, after which they must either return to duty or be evacuated to clearing stations.

Whether administered by a corps or a division, an entire field ambulance is immobilised for the management. The bearers, however, are available for duty at the Front in case of necessity. The patients, as soon as they have sufficiently recovered their strength, assist in the daily housework, and in many instances, where land is available, in the raising of vegetables—potatoes, cabbages, onions, and vegetable marrows being most popular, as they yield the most profitable return. These farms, initiated by field ambulances, are now becoming general among the units located on suitable land. A comprehensive scheme of considerable magnitude, drawn up by General Headquarters and supervised by a technical agricultural staff, has recently been introduced, continuity being ensured by a permanent supervising staff qualified to utilise continually changing labour.

The manufacture of "camouflage" is also undertaken by the patients. Each station has its own variety company formed from the R.A.M.C. staff, augmented, according to opportunity, by the patients, among whom there are frequently music-hall and other "stars," so that a first-class entertainment is the rule. Monotony is avoided by an interchange of performances with neighbouring units and by travelling companies and cinemas. Football, cricket, and sports provide outdoor amusement, and divisional or regimental bands attend frequently.

### Officers' Rest Stations.

The tired or shaken officer has not been forgotten; he also is provided with a haven of rest. Far from the firing-line in the château or town nearest to the safety limit, an institution on the lines of a Service club is open to suitable cases for a stay of fourteen days. Meals and drinks of the standard of an officers' mess are provided free of cost, and a reasonable amount of liberty is allowed. This institution is also managed by a detachment from a field ambulance.

### The Work of a Field Ambulance.

It will be gathered from the foregoing that the field ambulance must be one of the busiest units in the Expeditionary Force—such is an actuality—yet no other runs more smoothly and pleasantly, a great tribute to the organisation and staff work. There are twenty-four medical officers in the three field ambulances of a division, eight to each, distributed somewhat in the following manner:—

| | Officers. |
|---|---|
| Commanding officer, 1 each ... ... ... | 3 |
| Advanced dressing station and line of trenches of evacuation from regimental aid post, 2 each ... ... ... ... ... ... ... | 6 |
| One ambulance running a rest or scabies station ... ... ... ... ... ... | 3 |
| Officers' rest station ... ... ... ... | 1 |
| Two ambulances in the line receiving sick and wounded, 3 each ... ... ... ... ... | 6 |
| | 19 |

This leaves five officers to provide reliefs for the twenty-four field ambulance officers and about twenty unit medical officers, who have the privilege of proceeding on leave, if their services can be spared, every three months: to fill sudden vacancies caused by sickness or battle casualties; and to find operating teams for clearing stations.

This is manifestly a programme for the young and robust, and for them it is full of interest, excitement, and glory. The men also are equally busy; in addition to carrying and nursing the wounded and sick, they build their own huts and those for the sick and wounded, dig dug-outs in the trenches for the wounded and for themselves, and, if any spare moments remain, there is an eager demand for their services by other branches of the Service.

### Motor Ambulance Cars.

The slow, cumbrous horsed ambulance wagons of the beginning of the war have to a large extent been supplanted by motor cars. Nine of the former remain in each division, and they are used mainly for following troops on the move for picking up the foot-weary.

Each division now has twenty-one motor ambulance cars, including three "Fords." The "Fords" are wonders; they are used almost entirely for shell-hole dodging on the most advanced roads, which are impassable to the larger cars; they also attract less attention from sniping gunners.

These divisional cars are attached to field ambulances, and remove sick and wounded from advanced dressing stations and divisional areas to main dressing stations.

*(To be continued.)*

